# Renal cell carcinoma in the contralateral kidney with TFE3 gene translocation following chemotherapy for childhood nephroblastoma: A case report and literature review

**DOI:** 10.1002/ccr3.8128

**Published:** 2023-11-08

**Authors:** Shunsuke Fujisawa, Junya Furukawa, Takuto Hara, Keiske Okada, Kouji Chiba, Yuzo Nakano, Toshiki Hyodo, Yoji Nagashima, Masato Fujisawa

**Affiliations:** ^1^ Department of Urology Kobe University Graduate School of Medicine Kobe Japan; ^2^ Department of Diagnostic Pathology Kobe University Graduate School of Medicine Kobe Japan; ^3^ Division of Surgical Pathology Tokyo Women's Medical University Hospital Tokyo Japan

**Keywords:** case report, nephroblastoma, robot‐assisted partial nephrectomy, secondary malignant neoplasm, translocation renal cell carcinoma

## Abstract

**Key Clinical Message:**

Renal cell carcinoma as a secondary malignant neoplasm is relatively rare; however, the possibility of secondary renal cell carcinoma following chemoradiotherapy for childhood nephroblastoma should be considered.

**Abstract:**

The occurrence of secondary renal cell carcinoma (RCC) following chemoradiotherapy for nephroblastoma is relatively rare, especially in microphthalmia transcription factor family translocation renal cell carcinoma. A 13‐year‐old Japanese male was referred to our department for treatment of a right kidney mass. The patient had undergone open left nephrectomy and adjuvant chemotherapy for nephroblastoma, 12 years before. Diagnostic imaging revealed a tumor in the right kidney and a lesion suspected to be metastasis in the left eighth rib. Chromophobe RCC or translocation RCC was suspected from the imaging pattern. TNM classification was cT1aN0M1, and the clinical stage was IV. Partial nephrectomy by robot‐assisted surgery for the right renal tumor and resection of the left eighth rib were performed. Pathologically, the renal tumor was diagnosed as translocation RCC, and the rib lesion demonstrated no evidence of malignancy. We are currently undergoing imaging follow‐up and the patient has been recurrence‐free for 15 months. In this study, we present a rare case of secondary translocation RCC after successful treatment of nephroblastoma.

## 1 INTRODUCTION

Translocation renal cell carcinoma (RCC) is caused by genetic abnormalities such as TFE3 and was first defined in 2004.^1^ It is known that some patients of translocation RCC have a history of chemotherapy in childhood, but the underlying mechanism remains unclear.^2^ Although the occurrence of secondary malignant neoplasm (SMN) in association with chemoradiotherapy for nephroblastoma is firmly established, the incidence of secondary RCC is relatively rare, and the number of case reports of secondary translocation RCC is even more limited.^2^, ^3^ In this study, we present a rare case of contralateral secondary translocation RCC following open nephrectomy and postoperative chemoradiotherapy for nephroblastoma.

## 2 CASE PRESENTATION

A 13‐year‐old Japanese male was referred to our department for treatment of a right renal tumor that had been detected by abdominal ultrasound in a related pediatric clinic. The patient had been diagnosed with a large left kidney mass at the age of 11 months, and subsequently underwent an open left nephrectomy, resulting in a pathological diagnosis of nephroblastoma. The surgical and pathological findings indicated that the tumor was Stage III, and adjuvant chemoradiotherapy was administered, including total abdominal irradiation of 10.5 Gy and DD4A (a multidrug chemotherapy: vincristine/dactinomycin/doxorubicin). The last chemotherapy was administered in March 2010. In addition to the nephroblastoma, the patient was also diagnosed with two other urogenital malformations: hypospadias and right cryptorchidism. Following treatment for the nephroblastoma, urethroplasty and right orchiopexy were performed within 1 year after chemotherapy was completed.

**FIGURE 1:**
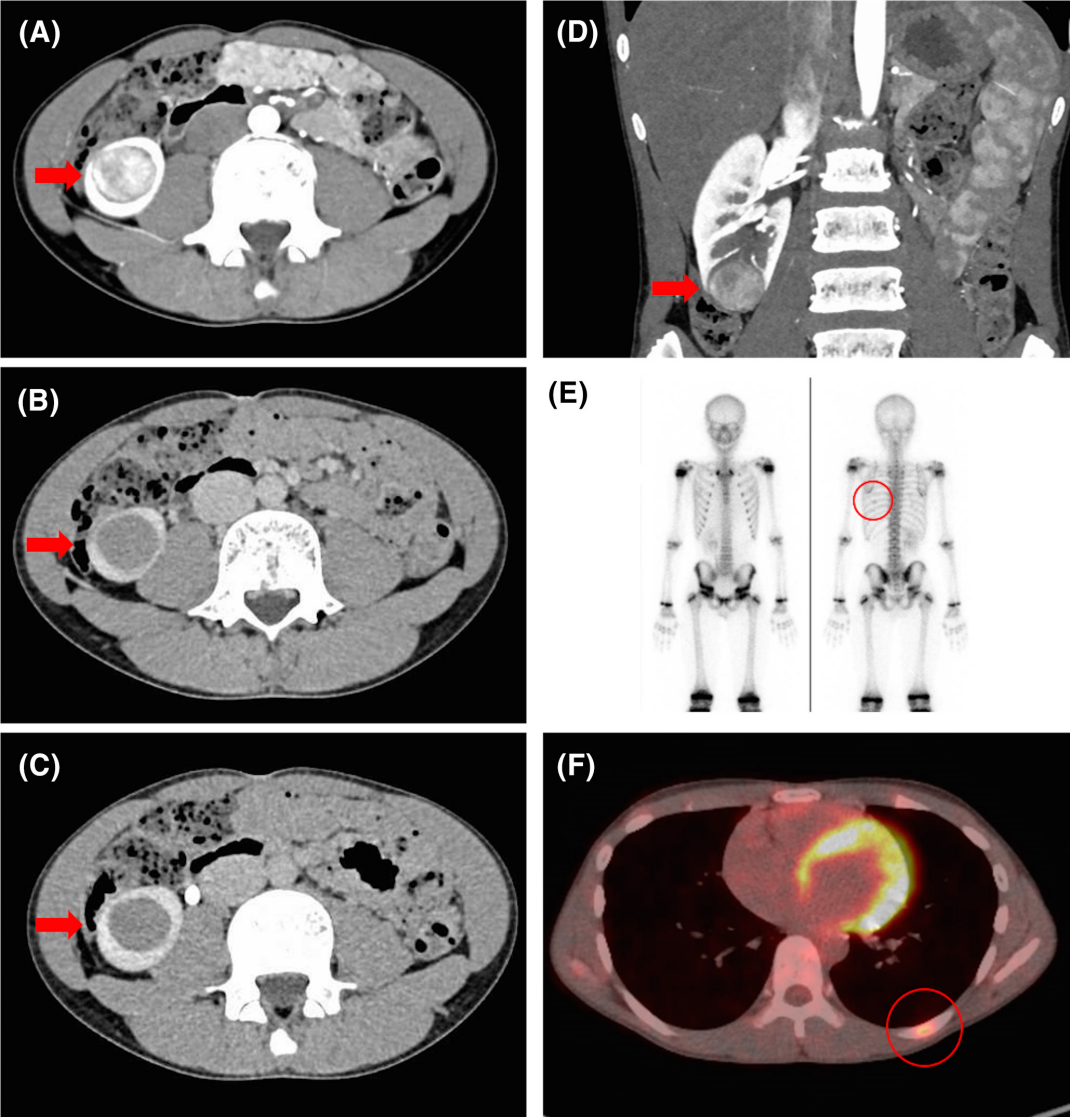
(A–D) Contrast‐enhanced CT showed a well‐defined 30 × 25 mm‐sized mass in the lower pole of the right kidney in the early phase, with a weak contrast effect below the renal cortex in the equilibrium phase and washout in the drainage phase (red arrow). (E, F) Bone scintigraphy and positron emission tomography‐CT showed an accumulation in the left eighth rib, which was suspected of metastasis (red circle).

**FIGURE 2:**
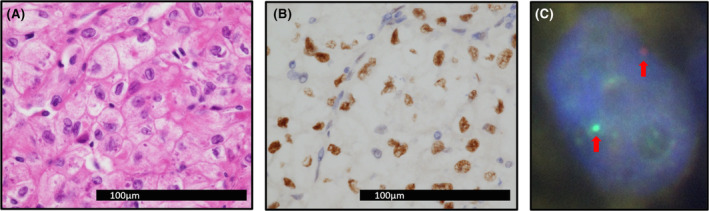
(A) The cells were eosinophilic, pale, and proliferating in a focal‐ and cord‐like structure, similar to clear cell RCC (×400 magnification). (B) Diffuse positive findings of TFE3 staining (×400 magnification). (C) Split signal pattern of *TFE3* break apart FISH (red arrow).

Enhanced computed tomography (CT) imaging revealed a 30‐mm‐sized mass in the lower pole of the right kidney. The mass was most strongly contrasted in the early phase, with a weaker contrast effect than the renal cortex in the equilibrium phase. Subsequently, contrast washout was observed in the drainage phase. The imaging pattern indicated chromophobe RCC or translocation RCC in the right kidney. The R.E.N.A.L. nephrometry score, which is a scoring system that categorizes the complexity of kidney tumors, was 8 points (1‐3‐3‐ × ‐1). Bone scintigraphy and positron emission tomography‐CT demonstrated an accumulation in the left eighth rib (Figure 1). The clinical diagnosis was right RCC with suspected single bone metastasis. TNM classification was cT1aN0M1, and the clinical stage was IV. It was hypothesized as being a secondary malignant neoplasm following nephroblastoma treatment. The patient's serum creatinine level at the time of initial consultation was 0.72 mg/dL. We decided to perform a partial nephrectomy by robot‐assisted surgery of the renal tumor for preservation of renal function and tumor resection of the left eighth rib. Given the patient's history of abdominal surgery, the procedure was performed via a retroperitoneal approach. A partial clamp of the renal artery associated with the tumor was performed during resection of the tumor. The operation time was 307 min, the console time was 176 min, and the warm ischemic time was 21 min. The amount of blood loss was minimal. The patient was discharged on postoperative day 8 without any complications. One month post‐surgery, the patient's serum creatinine concentration was 0.80 mg/dL. Pathological examination revealed that the tumor exhibited clear cell RCC characteristics on hematoxylin–eosin staining. However, carbonic anhydrase 9 staining was negative, which is atypical for clear cell RCC. Additional immunohistochemistry revealed positive transcription factor E3 (TFE3) staining, while *TFE3* fluorescence in situ hybridization (FISH) revealed a split signal (Figure 2). These examinations led to a definitive diagnosis of microphthalmia transcription factor family (MiT‐family) translocation RCC, Fuhrman nuclear grade 3, with no sarcomatous change, no lymphovascular invasion, and negative surgical margins. Resection of the left eighth rib was performed 3 months after partial nephrectomy. Histopathological findings demonstrated no evidence of malignancy (fibrotic lesion, lib). The patient is currently undergoing imaging follow‐up and has sustained no recurrence for 15 months.

## 3 DISCUSSION

MiT‐family translocation RCC has *TFE3* on chromosome Xp11.2 and the transcription factor *EB* gene on chromosome 6p21 as the major translocated genes.^4^, ^5^ The pathological feature of TFE3 translocation renal carcinomas is clear cells with solid to papillary architecture and frequent psammomatous calcifications.^6^ It is prevalent in younger people, and an association between a history of chemotherapy in childhood and the incidence has been reported.^2^

The mechanism by which RCC occurs secondarily after nephroblastoma treatment is not fully understood and is expected to have multiple components. Doxorubicin used in our patient and other DNA topoisomerase II inhibitors have been reported to cause double‐strand DNA breaks and DNA binding inhibition may cause chromosomal translocation, and it is quite possible that cytotoxic chemotherapy was a factor in this case.^2^

Brain tumors and basal cell carcinomas are well‐known SMNs after nephroblastoma treatment. However, RCC is relatively rare as them. A multi‐institutional study including over 13,000 cases of SMN after nephroblastoma treatment reported only 4 cases of secondary RCC.^2^ In our review of 15 cases of secondary RCC after nephroblastoma treatment including our study (Table 1, ^2^, ^7^, ^8^, ^9^, ^10^, ^11^, ^12^, ^13^, ^14^, ^15^), only 2 cases (13.3%) were diagnosed as translocation RCC.^2^ Eleven cases (73.3%) were clear cell RCC,^8^, ^9^, ^10^, ^11^, ^12^, ^13^, ^15^ 1 case (6.7%) was papillary cell RCC,^14^ and 1 case had an unspecified histologic type. In a review by Gupta et al. of 14 cases of secondary renal neoplasia occurring in patients treated with chemotherapy or radiation for nephroblastoma, acute lymphoblastic leukemia, neuroblastoma, and systemic lupus erythematosus, 9 cases were clear cell RCC and 2 cases were translocation RCC.^15^ These reviews suggest that the occurrence probability of translocation RCC as SMN is much lower than that of clear cell RCC. However, it might be underestimated. This is because translocation RCC was first classified in the 2004 World Health Organization classification of renal tumors^1^; prior cases were impossible to diagnose, and diagnosis of *TFE3* translocation RCC may be inaccurate in facilities that cannot perform the *TFE3* break apart FISH technique.^16^

The appropriate course of action for this clinical case was difficult to determine because of suspected metastasis. For a single metastatic RCC suspected in the left eighth rib, cytoreductive partial nephrectomy and resection of the metastases, or systemic therapy after tissue diagnosis by renal biopsy, are viable treatment options.^17^ However, given that this is a single kidney case, needle renal biopsy is a high‐risk option. Furthermore, the available evidence inadequately substantiates the effectiveness of immune checkpoint inhibitors or tyrosine kinase inhibitors in managing metastatic translocation RCC, although cabozantinib, a tyrosine kinase inhibitor targeting the highly expressed *MET* protein in translocation RCC, holds promise as a potential systemic therapeutic approach for this subtype of RCC.^18^ In our review, the longest recurrence‐free period of RCC after surgery was 22 months.^8^ Considering the need to preserve renal function, enhance the precision and safety of histological analysis, and ensure curative potential, partial nephrectomy was deemed the preferable option.

We performed a partial nephrectomy for the preservation of renal function. It has been postulated that factors associated with the preservation of postoperative renal function include younger age, higher preoperative estimated glomerular filtration rate, and greater preservation of renal parenchyma.^19^ Our patient fulfilled the above factors, which may have led to successful protection of postoperative renal function. In our review (Table 1, ^2^, ^7^, ^8^, ^9^, ^10^, ^11^, ^12^, ^13^, ^14^, ^15^), six cases underwent partial nephrectomy as surgical treatment.^8^, ^10^, ^11^, ^12^, ^13^ No reports of severe acute renal failure were observed during the operative period. These findings suggest that partial nephrectomy is also valuable in treating secondary RCC after nephroblastoma treatment with regard to the preservation of renal function.

**TABLE 1:** A review of patients with secondary renal cell carcinoma after treatment for nephroblastoma.

Patient No.	Reported year	Sex	Age at nephroblastoma diagnosis	Age at RCC diagnosis	Histology of RCC	Surgical treatment of RCC
1^7^	1977	F	3 years	24 years	Not reported	Radical nephrectomy and bench surgery
2^8^	1983	F	2 years	7 years	Clear cell with papillary pattern	Partial nephrectomy
3^9^	1994	F	1 years	32 years	Clear cell	Not reported
4^10^	2001	F	3 years	42 years	Clear cell	Heminephrectomy
5^10^	2001	M	3 years	40 years	Clear cell	Partial nephrectomy
6^11^	2005	F	2 years	31 years	Clear cell	Partial nephrectomy
7^2^	2006	Not reported	2 years	9 years	Translocation (*TFEB* fusion)	Not reported
8^12^	2009	F	4 years	18 years	Clear cell	Partial nephrectomy
9^13^	2010	F	4 years	17 years	Clear cell	Partial nephrectomy
10^14^	2017	F	5 years	47 years	Papillary	Not performed
11^15^	2020	Not reported	1 years	58 years	Clear cell	Not reported
12^15^	2020	Not reported	2 years	54 years	Clear cell	Not reported
13^15^	2020	Not reported	2 years	56 years	Clear cell	Not reported
14^15^	2020	Not reported	5 years	57 years	Clear cell	Not reported
Presented case	2023	M	11 months	13 years	Translocation (*TFE3* fusion)	Partial nephrectomy

Abbreviations: F, Female; M, Male.

## 4 CONCLUSION

We report a case of contralateral translocation RCC after treatment of nephroblastoma with radical resection by partial nephrectomy. This case implies that we need to recognize the possibility of RCC, including translocation RCC, as a secondary renal neoplasm after nephroblastoma treatment although RCC is a rare SMN following nephroblastoma treatment. Moreover, if the case is operable, partial nephrectomy can be considered for functional and oncological outcomes.

## AUTHOR CONTRIBUTIONS

**Shunsuke Fujisawa:** Writing – original draft. **Junya Furukawa:** Writing – review and editing. **Takuto Hara:** Writing – original draft. **Keiske Okada:** Writing – review and editing. **Kouji Chiba:** Writing – review and editing. **Yuzo Nakano:** Writing – review and editing. **Toshiki Hyodo:** Investigation. **Yoji Nagashima:** Investigation. **Masato Fujisawa:** Supervision.

## FUNDING INFORMATION

None.

## CONFLICT OF INTEREST STATEMENT

The authors declare no conflict of interest.

## ETHICS STATEMENT

None.

## PATIENT CONSENT STATEMENT

None.

## PERMISSION TO REPRODUCE MATERIAL FROM OTHER SOURCES

None.

## INFORMED CONSENT

Written informed consent was obtained from the patient for the publication of this case report.

## Data Availability

Data sharing is not applicable to this article as no new data were created or analyzed in this study.
