# Correction to “Renal cell carcinoma in the contralateral kidney with TFE3 gene translocation following chemotherapy for childhood nephroblastoma: A case report and literature review”

**DOI:** 10.1002/ccr3.9410

**Published:** 2024-11-07

Fujisawa S, Furukawa J, Hara T, et al. Renal cell carcinoma in the contralateral kidney with TFE3 gene translocation following chemotherapy for childhood nephroblastoma: a case report and literature review. *Clin Case Rep*. 2023;11(11):e8128.

One of coauthors' name is currently listed as “Keiske Okada,” but the correct spelling is “Keisuke Okada.”

We apologize for this error.

